# Association between lipoprotein(a) and premature atherosclerotic cardiovascular disease: a systematic review and meta-analysis

**DOI:** 10.1093/ehjopen/oeae031

**Published:** 2024-04-26

**Authors:** Xu Tian, Nan Zhang, Gary Tse, Guangping Li, Yihong Sun, Tong Liu

**Affiliations:** Tianjin Key Laboratory of Ionic-Molecular Function of Cardiovascular Disease, Department of Cardiology, Tianjin Institute of Cardiology, Second Hospital of Tianjin Medical University, Tianjin 300211, China; Tianjin Key Laboratory of Ionic-Molecular Function of Cardiovascular Disease, Department of Cardiology, Tianjin Institute of Cardiology, Second Hospital of Tianjin Medical University, Tianjin 300211, China; Tianjin Key Laboratory of Ionic-Molecular Function of Cardiovascular Disease, Department of Cardiology, Tianjin Institute of Cardiology, Second Hospital of Tianjin Medical University, Tianjin 300211, China; School of Nursing and Health Studies, Hong Kong Metropolitan University, Hong Kong, China; Epidemiology Research Unit, Cardiovascular Analytics Group, PowerHealth Limited, Hong Kong, China; Tianjin Key Laboratory of Ionic-Molecular Function of Cardiovascular Disease, Department of Cardiology, Tianjin Institute of Cardiology, Second Hospital of Tianjin Medical University, Tianjin 300211, China; Cardiology Department, China-Japan Friendship Hospital, Beijing, China; Tianjin Key Laboratory of Ionic-Molecular Function of Cardiovascular Disease, Department of Cardiology, Tianjin Institute of Cardiology, Second Hospital of Tianjin Medical University, Tianjin 300211, China

**Keywords:** Lipoprotein(a), Atherosclerotic cardiovascular disease, Risk factors, ‘Young’ patients, Meta-analysis

## Abstract

**Aims:**

High lipoprotein(a) [Lp(a)] level has been demonstrated as an important risk factor for atherosclerotic cardiovascular diseases (ASCVD) amongst the older populations, whereas its effects in the younger population remain unclear. This study evaluated the associations between Lp(a) and the risk of premature ASCVD.

**Method and results:**

PubMed and Embase were searched for related studies until 12 November 2023. Fifty-one studies including 100 540 participants were included. Mean age of patients ranged from 35.3 to 62.3 years. The proportion of male participants ranged from 0% to 100%. The mean follow-up was provided in five studies ranging from 1 year to 40 years. The definition of elevated Lp(a) varied among studies, such as >30 mg/dL, >50 mg/dL, the top tertiles, the top quartiles, the top quintiles, and so on. Higher Lp(a) was significantly associated with the composite ASCVD [odds ratio (OR): 2.15, 95% confidence interval (95% CI): 1.53–3.02, *P* < 0.001], especially for coronary artery disease (OR: 2.44, 95% CI: 2.06–2.90, *P* < 0.001) and peripheral arterial disease (OR: 2.56, 95% CI: 1.56–4.21, *P* < 0.001). This association remained significant in familial hypercholesterolaemia (FH) (OR: 3.11, 95% CI: 1.63–5.96, *P* < 0.001) and type 2 diabetes mellitus (T2DM) patients (OR: 2.23; 95% CI: 1.54–3.23, *P* < 0.001).Significant results were observed in South Asians (OR: 3.71, 95% CI: 2.31–5.96, *P* < 0.001), Caucasians (OR: 3.17, 95% CI: 2.22–4.52, *P* < 0.001), and patients with baseline low-density lipoprotein cholesterol (LDL-c) level ≥ 2.6 mmol/L.

**Conclusion:**

Elevated Lp(a) predicts the risk of the composite or individual ASCVD in young, regardless of study design, gender, population characteristics (community or hospitalized), different premature definitions, and various Lp(a) measurement approaches. This association was important in South Asians, Caucasians, FH patients, T2DM patients, and patients with baseline LDL-c level ≥ 2.6 mmol/L.

## Graphical Abstract

**Graphical Abstract:**
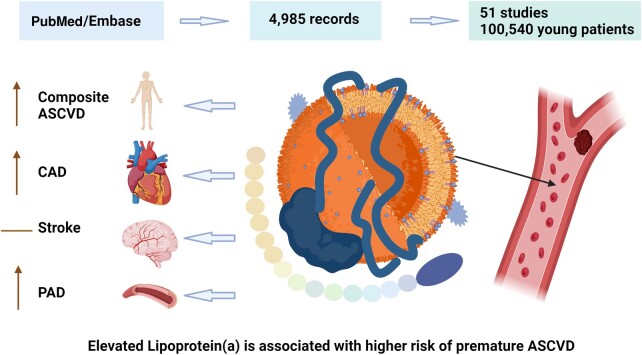

## Introduction

Atherosclerotic cardiovascular disease (ASCVD) remains the leading cause of death worldwide, with an estimated annual global mortality rate of 17.9 million patients.^1,2^ Although the majority of ASCVD and associated adverse events occurs among the elderly, the younger population also remains vulnerable.^3^ The prevalence of ASCVD in those under the age of 60 has been reported to range from 7% to 30% depending on different domains of ASCVD and geographic regions.^1^ Atherosclerotic cardiovascular disease events in young patients may have an important impact on the patient’s longevity, psychology, and socioeconomics, leading to huge disability-adjusted life lost, as well as heavy healthcare economic burdens on society.^3^

Recently, lipoprotein(a) [Lp(a)], a low-density lipoprotein cholesterol (LDL-c)-like particle, has been demonstrated as a novel risk factor for various cardiovascular outcomes, such as ASCVD and aortic valve stenosis.^4^ However, previous studies regarding the association between Lp(a) and ASCVD have mainly focused on the older or general populations.^5^ There is a scarcity of evidence on the association between Lp(a) and ASCVD among young patients. The BIOSIGNAL study has observed a significant association between elevated Lp(a) and large artery atherosclerosis (LAA) stroke amongst individuals aged <60 years.^6^ A prospective study of 3596 patients by Raitakari *et al.*^7^ also found that higher Lp(a) was related to premature ASCVD, whereas Cai *et al.*^8^ and Shi *et al.*^9^ reported no significant relationship between Lp(a) and acute myocardial infarction (AMI) at the early age.

There was a previous meta-analysis showing that elevated Lp(a) was associated premature coronary artery disease (CAD). However, they only included 11 studies with moderate quality, in which the sample size was relatively small. High statistical heterogeneity was also the main limitation of the meta-analysis. In addition, the role of higher Lp(a) in the stroke and peripheral artery disease (PAD) has not been studied. Given the discrepant findings from the existing studies, we conducted a systematic review and meta-analysis to clarify the relationship between Lp(a) and premature ASCVD.

## Methods

### Study design and search strategy

The meta-analysis was performed according to the Preferred Reporting Item for Systematic Reviews and Meta-Analyses statement.^10^ The study protocol was registered in the PROSPERO database on 12 November 2023 (registration number: CRD42023476725). PubMed and EMBASE were searched from their inception through 26 October 2023. We used both controlled MeSH terms and free-text terms related to premature ASCVD and Lp(a) to identify related studies, with no language restrictions. The detailed search algorithm is presented in the Supplementary material online. References listed in the identified studies were scrutinized for relevant studies. Trial eligibility was confirmed by two independent reviewers (X.T. and N.Z.), and discrepancies were resolved by a third author (T.L.).

### Outcome and selection criteria

The outcome was the occurrence of any ASCVD at the age <65.^11–14^ Atherosclerotic cardiovascular disease in this study included CAD, stroke, and PAD.^15–17^ A study was eligible if the following criteria were fulfilled: (i) observational studies conducted in humans; (ii) investigating the associations between blood Lp(a) level and premature ASCVD; (iii) blood Lp(a) level was reported as a continuous (e.g. per one unit or per one standard deviation (SD) increase of Lp(a) or log [Lp(a)]) or categorical variable (e.g. tertile, quartile, quintile, or specific thresholds); and (iv) reported the composite ASCVD or at least one individual outcome. The exclusion criteria were (i) comments, reviews, or animal studies, (ii) studies not investigated Lp(a) level, (iii) age of onset was ineligible, (4) outcomes with interest were not reported; and (v) helpful odds ratio (OR)/risk ratio (RR)/hazard ratio (HR) were not provided.

### Data extraction

Two reviewers (X.T. and N.Z.) independently performed data extraction. Pre-specified forms were used for data collection to include the following items: (i) publication details: first author, publication year, country, and study design; (ii) baseline characteristics: sample size, age of onset, duration of follow-up, and outcome-related variables; and (iii) Lp(a) measurements. Conflicts between investigators were resolved by discussion and, if necessary, through consultation with a third reviewer (T.L.).

### Quality assessment

Quality of cohort studies and case-control studies was assessed by the Newcastle-Ottawa Quality Assessment Scale (NOS) from the following three aspects: (i) selection of patients, (ii) comparability of groups, and (iii) ascertainment of exposure or outcomes.^18^ Varying from zero to nine stars, the studies were graded as high if they met ≥8 criteria, medium if they met 5–7 criteria, and poor if they met <5 criteria. Quality assessment of cross-sectional studies was performed using the American Agency for Healthcare Research and Quality (AHRQ) criterion with 11 items.^19^ The AHRQ varied from 0 to 11 stars, which indicated that studies were graded as poor quality if they met <5 criteria, fair if they met 6–7 criteria, and good if they met ≥8 criteria.

### Statistical analysis

Hazard ratio, RR, OR, and corresponding 95% confidence interval (CI) were extracted from the fully adjusted models to evaluate the of association between Lp(a) and outcomes.^20^ When results of multivariable analysis were not available, those from univariable analysis were used.^21^ If HR/RR/OR was not reported, event number in the case and control groups was applied to calculate the unadjusted risk estimates.^22^ The HR/RR value in Cox proportional hazards model was equated to the OR value.^23,24^ Pooled effects were summarized as OR (95% CI) using the inverse variance method.^25^ Heterogeneity was assessed by using the Cochrane *Q* statistic and *I*^2^ statistic. For the *Q* test, *P* value < 0.1 was considered statistically significant. *I*^2^ < 50% was considered as no heterogeneity and <70% as moderate heterogeneity, otherwise as high. If *I*² value > 50%, the random-effects model was used; otherwise, the fixed-effects model was used. The ORs are scaled per one unit or SD change of Lp(a) or log [Lp(a)] for continuous variable and compared with the lowest quantile for categorical variable. Lp(a) could be reported in the mass concentration method (mg/dL) and the molar concentration method, and no fixed value was used as a conversion factor between the two units.^26^ However, to calculate the data of different units, levels above 72 nmol/L were considered consistent with traditional thresholds for elevated Lp(a) above 30 mg/dL,110 nmol/L for 50 mg/dL, and 450 nmol/L for 180 mg/dL. When the definition of elevated Lp(a) was the top tertile (compared with the bottom tertile), the top quartile (compared with the bottom quartile), and the top quintile (compared with the bottom quintile), the difference of unit did not affect data merging.^27^

Subgroup analyses were performed according to study design, gender, race (Caucasian, South Asian, and Chinese), population characteristics (community population and hospitalization population), baseline LDL-c level [<100 mg/dL (2.6 mmol/L), 100–130 mg/dL (2.6–3.4mmo/L), 130–160 mg/dL (3.4–4.1 mmol/L), and ≥160 mg/dL (4.1 mmol/L)],^28^ definition of the ‘premature’ (50 years and 60 years),^14^ categorization approaches of Lp(a) [tertile, quartile, quintile, or specific cut-off such as 30 mg/dL (72 nmol/L) and 50 mg/dL (110 nmol/L)], and Lp(a) measurements [enzyme-linked immunosorbent assay (ELISA), immunoturbidimetric method, and immunoradiometric assay (IRMA)]. Due to the limited number of studies that reported continuous variables, subgroup analyses were mainly conducted using categorical variables. To evaluate the impact of each study on the overall effect size, one study which removed sensitivity analysis was performed. Funnel plots used to assess possible publication bias. Statistical significance was defined as *P* values < 0.05. Statistical analyses were performed with the Review Manger, version 5.4 (RevMan; The Cochrane Collaboration, Oxford, UK).

## Results

### Study selection and study characteristics

**Figure 1:**
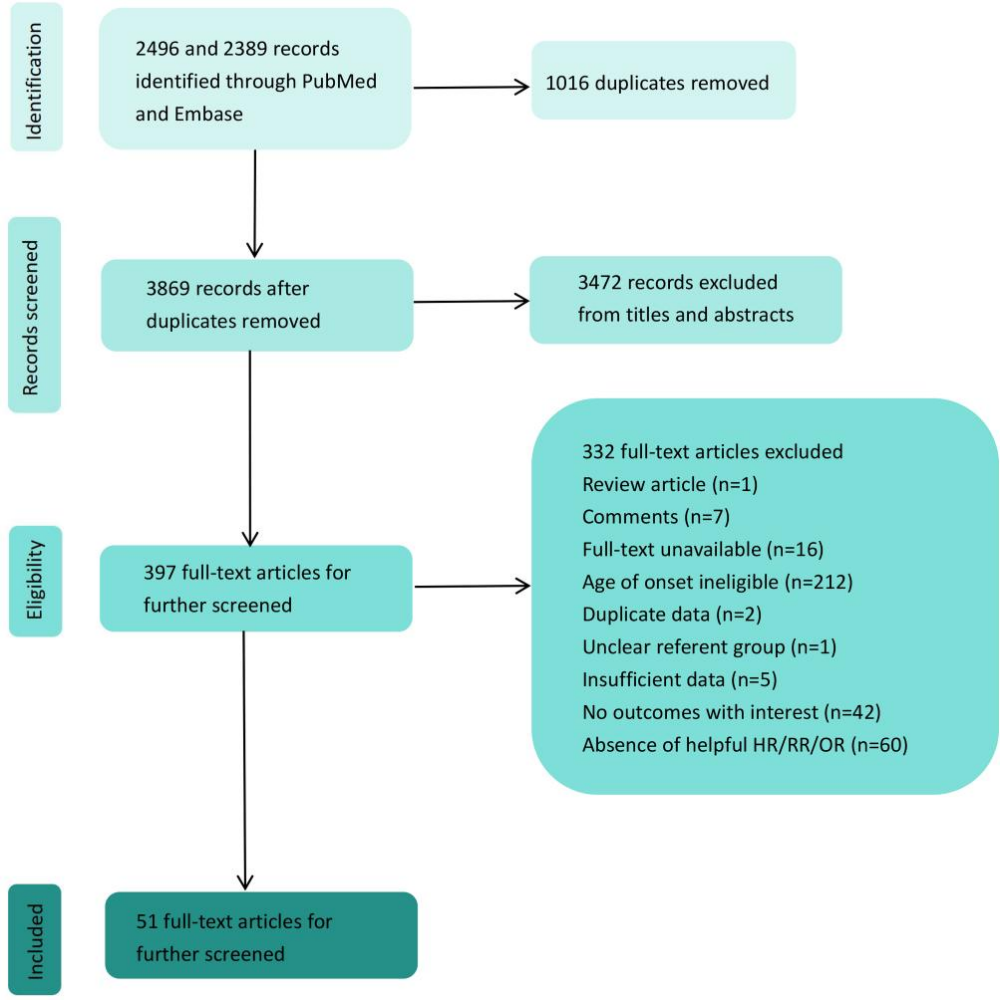
Flow diagram of the study selection process.

Initially, a total of 2496 and 2489 records were identified through PubMed and Embase, and 51 studies were finally included in the meta-analysis. A flow diagram of the data search and study selection is shown in *Figure 1*. The baseline characteristics of the included studies are summarized in *Table 1*. A total of 51 studies including 100 540 individuals were studied, of which 16 were cross-sectional studies, 9 were prospective cohort studies, and 26 were retrospective studies. The proportion of male participants ranged from 0% to 100%. The mean follow-up was provided in five studies ranging from 1 year to 40 years. Nineteen studies included hospital-based patients, and others included community-based patients. Quality assessment indicated that all of included studies were graded as high or medium quality.

**Table 1:** Characteristics of the 51 studies included in the meta-analysis

Frist author and year	Country	Study design	Study population	Total patients	Males (%)	Race	Baseline ASCVD (%)	Age of onset	Follow-up duration	Technique of Lp(a) measurement	Baseline LDL-c level (mmol/L)	Outcomes	Risk estimate	Quality score
Aasvee 2006	Estonia	Case-control	Community-based	156	156 (100.0)	NA	NA	≤55 yrs	NA	IRMA	3.4–4.1	MI	OR	7
Anagnostis 2022	Greece	Cross-sectional	Community-based	541	249 (46.0)	NA	NA	M: <55 yrs, F: <60 yrs	NA	NA	≥4.1	CAD, ASCVD	OR	7
Arnold 2021	Switzerland	Prospective cohort	Hospital-based	339	NA	White	339 (100)	<60 yrs + 1 year	1 year	Roche Tinaquant assay	NA	Stroke	HR	9
Be'rard 2013	France	Case-control	Community-based	305	236 (77.4)	NA	12 (3.9)	<45 yrs	NA	Nephelometery	2.6–3.4	PAOD	OR	8
Beheshtian 2016	USA	Prospective nested case-control	Community-based	645	365 (56.6)	White: 398, Black: 78, Hispanic: 115, Asian: 36, Other: 18	NA	18–64yrs	NA	Immunoturbidimetric assay	NA	Stroke	OR	8
Dahlén 1998	Sweden	Case-control	Community-based	102	0 (0.0)	White: 45, Black: 57	NA	≤65 yrs	NA	ELISA	≥4.1	CAD	OR	9
Dugani 2021	USA	Prospective cohort	Community-based	26 539	0 (0.0)	White: 26 475, Black: 520, Hispanic: 290, Asian: 382, Other: 119	NA	<55 yrs	21.4 yrs	NA	NA	CHD	HR	9
Elisaf 1997	Greece	Case-control	Community-based	212	169 (14.1)	NA	NA	M: <55 yrs, F: <65 yrs	NA	ELISA	3.4–4.1	CAD	OR	9
Ellis 2018	Australia	Cross-sectional	Hospital-based	316	221 (69.9)	NA	NA	<60 yrs	NA	Latex-enhanced turbidimetric immunoassay	2.6–3.4	CAD	OR	8
Foody 2000	USA	Cross-sectional	Hospital-based	822	556 (67.6)	NA	NA	20–55 yrs	NA	Immunoturbidimetric assay	NA	CAD	OR	7
Gambhir 2008	India	Case-control	Community-based	380	329 (86.6)	South Asian	NA	<40 yrs	NA	ELISA	2.6–3.4	CAD	OR	8
George 2016	India	Case-control	Community-based	254	204 (80.3)	South Asian	NA	≤55 yrs	NA	Latex-enhanced turbidimetric immunoassay	2.6–3.4	CAD	OR	8
Hanif 2019	Pakistan	Case-control	Community-based	90	NA	NA	NA	≤45 yrs	NA	Latex-enhanced turbidimetric immunoassay	NA	CAD	OR	7
Hopkins 1997	USA	Case-control	Community-based	335	209 (62.4)	NA	NA	M: <55 yrs, F: <65 yrs	NA	ELISA	3.4–4.1	CAD	OR	6
Hopkins 2001	USA	Case-control	Community-based	262	112 (42.7)	NA	NA	M: <55 yrs, F: <65 yrs	NA	ELISA	NA	CAD	OR	8
Joseph 2022	India	Case-control	Hospital-based	186	164 (88.2)	South Asian	NA	<50 yrs	NA	Immunoturbidimetric method	3.4–4.1	CAD	OR	8
Jubran 2019	Israel	Retrospective cohort	Hospital-based	134	111 (83.0)	NA	5 (4)	M: <55 yrs, F: <60 yrs	NA	Immunoturbidimetric method	2.6–3.4	CAD	OR	7
Klausen 1997	Denmark	Prospective nested case-control	Community-based	160	160 (100)	NA	NA	<60 yrs	NA	IRMA	NA	CHD	OR	9
Lan 2021	Australia	Cross-sectional	Hospital-based	449	328 (73.1)	NA	NA	<60 yrs	NA	Latex-enhanced turbidimetric immunoassay	2.6–3.4	CAD	OR	8
Li 2017	China	Cross-sectional	Hospital-based	4346	NA	Chinese	NA	M: <55 yrs, F: <60 yrs	NA	Immunoturbidimetry method	NA	CAD	OR	8
Longenecker2003	USA	Cross-sectional	Hospital-based	436	NA	White: 261, Black: 150	NA	<60 yrs	NA	ELISA	NA	ASCVD	OR	7
Marcucci 2005	Italy	Case-control	Community-based	280	226 (80.7)	NA	NA	M: ≤50 yrs, F: ≤55 yrs	NA	ELISA	NA	CAD	OR	7
Orth-Gomer1997	Sweden	Case-control	Community-based	484	0 (0.0)	NA	NA	≤65 yrs	NA	Immunoturbidimetric method	≥4.1	CHD	OR	9
Pineda 2009	Spain	Case-control	Community-based	237	219 (92.4)	NA	NA	≤45 yrs	NA	NA	2.6–3.4	CAD	OR	8
Raitakari 2023	Helsinki	Prospective cohort	Community-based	3596	NA	NA	NA	31–56 yrs	40 yrs	IRMA	NA	ASCVD	HR	9
Rallidis 2018	Greece	Case-control	Community-based	1482	944 (63.7)	NA	NA	<45 yrs	NA	Immunonephelometry	NA	ACS	OR	8
Reibis 2012	Germany	Cross-sectional	Hospital-based	15 381	10 182 (66.2)	NA	NA	M: <55 yrs, F: <65 yrs	NA	NA	NA	CAD	OR	7
Rigal 2007	France	Prospective nested case-control	Community-based	200	116 (58.0)	NA	NA	18–55 yrs	NA	Immunoturbidimetric method	M: 3.4–4.1, F: 2.9–3.4	Stroke	OR	7
Shi 2021	China	Cross-sectional	Hospital-based	2433	1157 (47.6)	Chinese	NA	M: <55 yrs, F: <65 yrs	NA	ELISA	<2.6	CAD	OR	8
Sunayama 1996	Japan	Case-control	Hospital-based	84	0 (0.0)	NA	NA	≤55 yrs	NA	ELISA	2.6–3.4	CAD	OR	6
Tsimikas 2005	USA	Cross-sectional	Hospital-based	239	NA	NA	77 (15)	≤60 yrs	NA	Commercially available kits	2.6–3.4	CAD	OR	8
Valentine 1996	USA	Case-control	Community-based	95	95 (100.0)	White	NA	≤45 yrs	NA	ELISA	NA	PVD	OR	8
Wei 2022	China	Cross-sectional	Hospital-based	2846	1397 (49.1)	NA	NA	M: <45 yrs, F: <55 yrs	NA	NA	2.6–3.4	CHD	OR	8
Zorio 2006	Spain	Case-control	Community-based	421	377 (89.5)	NA	NA	<51 yrs	NA	ELISA	2.6–3.4	MI	OR	7
Tyurian 2023	UK	Case-control	Community-based	300	NA	NA	NA	M: <55 yrs, F: <60 yrs	NA	NA	NA	CHD	RR	7
Shi 2023	China	Cross-sectional	Hospital-based	1626	1213 (74.6)	Chinese	NA	M: <55 yrs, F: <65 yrs	NA	Immunoturbidimetric method	2.6–3.4	MI	OR	8
Ezhov 2023	Russia	Cross-sectional	Community-based	8461	3072 (36.3)	NA	NA	25–64 yrs	NA	Immunoturbidimetric method	2.6–3.4	CHD, MI, stroke	OR	7
Tyurina 2022	Russia	Case-control	Community-based	228	228 (100)	NA	46 (20.2)	<55 yrs	NA	NA	2.6–3.4	CAD	OR	8
McCaughey 2022	Ireland	Case-control	Hospital-based	109	92 (84.4)	NA	NA	≤45 yrs	NA	NA	NA	MI	OR	5
Simony 2022	USA	Prospective cohort	Community-based	18 034	7806 (43.2)	NA	NA	<50 yrs	NA	NA	NA	MI, IHD, stroke	HR	8
Chubykina 2022	Russia	Cross-sectional	Community-based	164	123 (75)	NA	NA	<60 yrs	NA	ELISA	<2.6	ACS	OR	8
Cai 2021	China	Cross-sectional	Hospital-based	1040	NA	Chinese	NA	<60 yrs	NA	Immunoturbidimetric method	NA	MI	OR	8
Rallidis 2017	Greece	Case-control	Community-based	500	NA	NA	NA	<35 yrs	NA	NA	NA	MI	OR	7
Shai 2005	USA	Prospective cohort	Community-based	921	0(0)	NA	NA	30–55 yrs + 10 yrs	10 yrs	Immunoturbidimetric method	3.4–4.1	CHD	OR	9
García-Díaz 2003	NA	Case-control	Community-based	78	NA	NA	NA	<65 yrs	NA	NA	NA	MI	OR	7
Li Wang 2000	Australia	Cross-sectional	Hospital-based	1308	995 (78.1)	NA	NA	≤65 yrs	NA	ELISA	M: 2.9–3.4, F: 3.4–4.1	CAD	OR	7
Solymoss 1993	Canada	Cross-sectional	Hospital-based	274	0 (0)	White	NA	≤60 yrs	NA	NA	3.4–4.1	CAD	OR	8
Bostom 1996	USA	Prospective cohort	Community-based	2191	2191 (100)	NA	NA	≤55 yrs	15.4 yrs	ELISA	NA	CHD, MI	RR	8
Cao 2003	China	Case-control	Hospital-based	93	NA	Chinese	NA	≤45 yrs	NA	Immunoturbidimetric method	<2.6	Stroke	OR	8
Gambhir 2000	India	Case-control	Community-based	100	NA	NA	NA	<40 yrs	NA	ELISA	NA	CAD	OR	7
Wityk 2000	USA	Case-control	Community-based	326	0 (0)	White: 202, Black: 116, Other:8	22 (6.7)	<45 yrs	NA	ELISA	NA	Stroke	OR	8

ACS, acute coronary syndrome; ASCVD, arteriosclerotic cardiovascular disease; CAD, coronary artery disease; CHD, coronary heart disease; ELISA, enzyme-linked immunosorbent assay; HR, hazard ratio; IRMA, immunoradiometric assay; LDL-c, low-density lipoprotein- cholesterol; MI, myocardial infarction; NA, not available; OR, odds ratio; PAOD, peripheral arterial occlusive disease; PVD, peripheral vascular disease

### Association between lipoprotein(a) and premature atherosclerotic cardiovascular diseases

#### Lipoprotein(a) and overall atherosclerotic cardiovascular diseases among overall population

**Figure 2:**
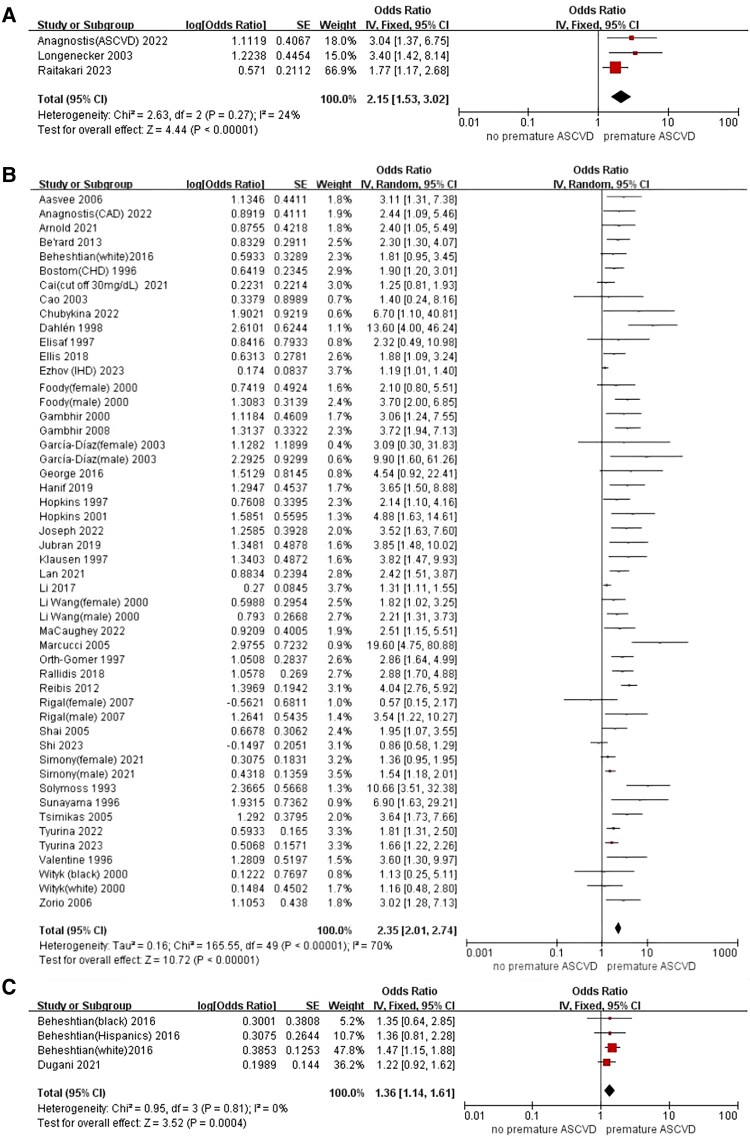
Meta-analysis of elevated Lp(a) and the risk of the composite ASCVD at the early age (*A*). Meta-analysis of elevated Lp(a) and the risk of the individual ASCVD at the early age for categorical variables (*B*), and continuous variable as per 1 SD increase of the log Lp(a) (*C*). ASCVD, arteriosclerotic cardiovascular disease; CI, confidence interval; Lp(a), lipoprotein(a); OR, odds ratio; SE, standard error.

A total of three studies reporting the association between Lp(a) and composite ASCVD, which suggested that compared with lower Lp(a), elevated Lp(a) level was associated with significant higher risks of composite ASCVD events among individuals at an early age (OR: 2.15, 95% CI: 1.53–3.02, *P* < 0.001) (*Figure 2A*). In addition, after pooling 49 studies focusing on individual ASCVD events, the association between increased Lp(a) level and overall ASCVD remained significant when analysed as a categorical variable (OR: 2.35, 95% CI: 2.01–2.74, *P* < 0.001) (*Figure 2B*) or continuous variable as per 1 SD increase of the log Lp(a) (OR: 1.36, 95% CI: 1.14–1.61, *P* < 0.001) (*Figure 2C*) but not for other forms of continuous variable (see Supplementary material online, *Figure S2*).

#### Lipoprotein(a) and overall atherosclerotic cardiovascular diseases among specific populations

**Figure 3:**
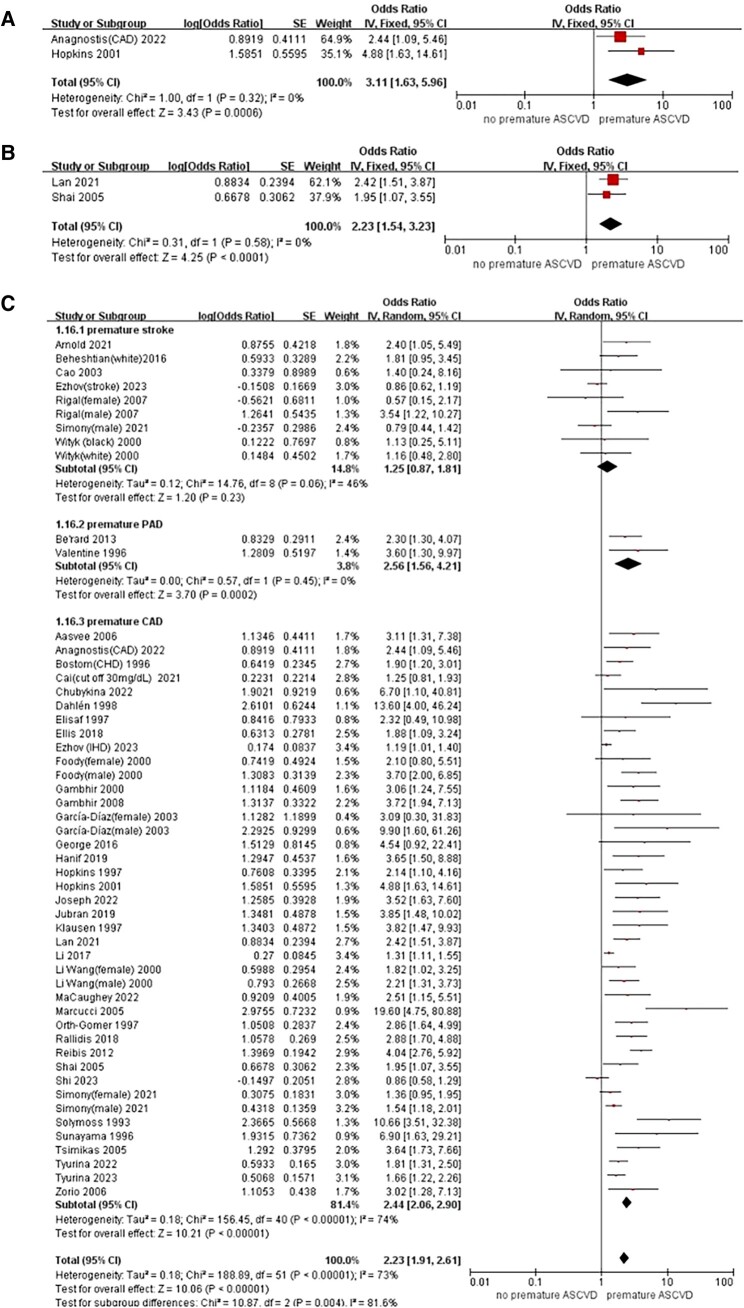

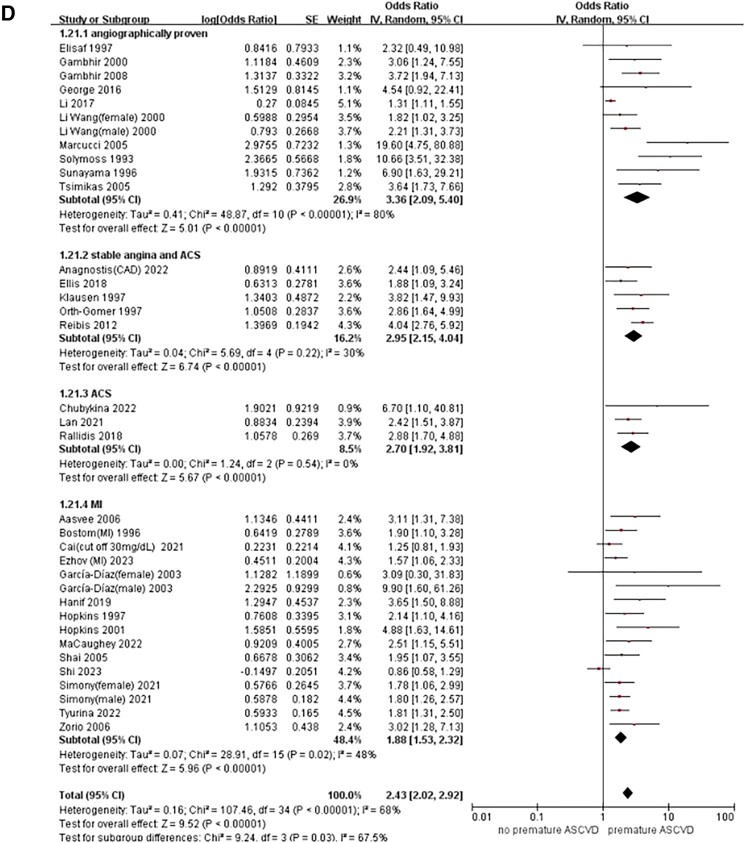
Further analysis of elevated Lp(a) and the risk of overall ASCVD at the early age in FH patients (*A*) and in T2DM patients (*B*) when analysed as categories. Meta-analysis of elevated Lp(a) and the risk of individual ASCVD at the early age (*C*) and further analysis classifying premature CAD into subcategories (*D*). ASCVD, arteriosclerotic cardiovascular disease; CAD, coronary artery disease; CI, confidence interval; FH, familial hypercholesterolaemia; Lp(a), lipoprotein(a); OR, odds ratio; PAD, peripheral arterial disease; SE, standard error; T2DM, type 2 diabetes mellitus.

In addition, the aggregated data of three studies showed that significant prognostic value of Lp(a) level for premature ASCVD was significant in familial hypercholesterolaemia (FH) patients (OR: 3.11, 95% CI: 1.63–5.96, *P* < 0.001) (*Figure 3A*). A total of two studies focused on patients with type 2 diabetes mellitus; the pooled results of these two studies suggested a significant association between elevated Lp(a) with premature ASCVD events among T2DM population (OR: 2.23; 95% CI: 1.54–3.23, *P* < 0.001) (*Figure 3B*), in relation to those with lower Lp(a). Funnel plot showed little publication bias (see Supplementary material online, *Figure S1*).

#### Lipoprotein(a) and individual atherosclerotic cardiovascular diseases

As for the individual ASCVD events, there was a positive association between Lp(a) levels with risk of premature CAD (OR: 2.44, 95% CI: 2.06–2.90, *P* < 0.001) and PAD (OR: 2.56, 95% CI: 1.56–4.21, *P* < 0.001) but not for stroke (OR: 1.25, 95% CI: 0.87–1.81, *P* = 0.06) (*Figure 3C*). When premature CAD was further classified into subcategories, the association remained significant between elevated Lp(a) level and increased risk of angiographically proven CAD (OR: 3.36, 95% CI: 2.09–5.40, *P* < 0.001), stable angina and ACS (OR: 2.95, 95% CI: 2.15–4.04, *P* < 0.001), ACS only (OR: 2.70, 95% CI: 1.92–3.81, *P* < 0.001), as well as MI (OR: 1.88, 95% CI: 1.53–2.32, *P* < 0.001) (*Figure 3D*).

### Subgroup analyses

The results of subgroup analysis were reported in *Table 2*. In subgroup analysis according to study design, elevated Lp(a) was associated with significantly increased risk of premature ASCVD in cross-sectional studies (OR: 2.09, 95% CI: 1.59–2.75, *P* < 0.001), retrospective studies (OR: 2.84, 95% CI: 2.31–3.50, *P* < 0.001), and prospective studies (OR: 1.73, 95% CI: 1.40–2.13, *P* < 0.001) (see Supplementary material online, *Figure S3*). In addition, the association between Lp(a) and premature ASCVD was significant both in male (OR: 2.31, 95% CI: 1.80–2.97, *P* < 0.001) and female patients (OR: 2.34, 95% CI: 1.53–3.58, *P* < 0.001) (see Supplementary material online, *Figure S4*). As for race, elevated Lp(a) could predict premature ASCVD in South Asians (OR: 3.71, 95% CI: 2.31–5.96, *P* < 0.001) and white patients (OR: 3.17, 95% CI: 2.22–4.52, *P* < 0.001) but not in Chinese (OR: 1.20, 95% CI: 0.99–1.45, *P* = 0.06) (see Supplementary material online, *Figure S5*). In subgroup analysis by population characteristics, the significant association between higher Lp(a) and increased risk of premature ASCVD was noted both among community population (OR: 2.34, 95% CI: 1.93–2.84, *P* < 0.001) and hospitalized patients (OR: 2.37, 95% CI: 1.78–3.15, *P* < 0.001) (see Supplementary material online, *Figure S6*).

**Table 2:** Subgroup analysis of the association between elevated lipoprotein(a) and the risk of premature atherosclerotic cardiovascular diseases for categorical variables

	Subgroup	Number of studies	Meta-analysis			Heterogeneity	
			OR	95% CI	*P* value	*I*² (%)	*P* value
Study design	Prospective	9	1.73	1.40–2.13	*P* < 0.001	22	*P* = 0.25
	Retrospective	26	2.84	2.31–3.50	*P* < 0.001	41	*P* = 0.02
	Cross-sectional	15	2.09	1.59–2.75	*P* < 0.001	83	*P* < 0.001
Gender	Female	12	2.34	1.53–3.58	*P* < 0.001	66	*P* = 0.006
	Male	10	2.31	1.80–2.97	*P* < 0.001	44	*P* = 0.06
Race	Caucasians	11	3.17	2.22–4.52	*P* < 0.001	51	*P* = 0.03
	South Asians	3	3.71	2.31–5.96	*P* < 0.001	0	*P* = 0.96
	Chinese	4	1.20	0.99–1.45	*P* = 0.06	17	*P* = 0.31
Population characteristics	Community population	32	2.34	1.93–2.84	*P* < 0.001	65	*P* < 0.001
	Hospitalized patients	18	2.37	1.78–3.15	*P* < 0.001	78	*P* < 0.001
Baseline LDL-c level	<2.6 mmol/L	2	3.02	0.65–14.00	*P* = 0.16	32	*P* = 0.22
	2.6–3.4 mmol/L	12	2.09	1.56–2.79	*P* < 0.001	75	*P* < 0.001
	3.4–4.1 mmol/L	9	2.70	1.92–3.80	*P* < 0.001	30	*P* = 0.19
	≥4.1 mmol/L	4	4.38	2.57–7.46	*P* < 0.001	44	*P* = 0.15
Definition of ‘premature’	60 years	34	2.40	2.00–2.89	*P* < 0.001	63	*P* < 0.001
	50 years	12	2.72	2.16–3.41	*P* < 0.001	0	*P* = 0.71
Different cut-off levels of Lp(a)	Cut-off 30 mg/dL	18	2.19	1.75–2.74	*P* < 0.001	65	*P* < 0.001
	Cut-off 50 mg/dL	5	2.98	1.94–4.58	*P* < 0.001	56	*P* = 0.06
	Quintiles	4	1.47	0.78–2.76	*P* = 0.23	75	*P* = 0.02
	Quartiles	9	2.39	1.55–3.68	*P* < 0.001	65	*P* = 0.004
	Tertiles	3	2.22	1.15–4.29	*P* = 0.02	43	*P* = 0.15
Technique of Lp(a) measurement	ELISA	16	2.93	2.15–3.98	*P* < 0.001	48	*P* = 0.02
	Immunoturbidimetric method	18	1.86	1.50–2.31	*P* < 0.001	69	*P* < 0.001
	IRMA	2	3.41	1.80–6.48	*P* < 0.001	0	*P* = 0.75

ACS, acute coronary syndrome; ASCVD, atherosclerotic cardiovascular diseases; CAD, coronary artery diseases; CI, confidence interval; ELISA, enzyme-linked immunosorbent assay; IRMA, immunoradiometric assay; Lp(a), lipoprotein(a); LDL-c, low-density lipoprotein cholesterol; MI, myocardial infarction; OR, odds ratio.

As for the baseline LDL-c level, the association between elevated Lp(a) level and increased risk of premature ASCVD was significant among patients with baseline LDL-c level of 100–130 mg/dL (2.6–3.4 mmol/L) (OR: 2.09, 95% CI: 1.56–2.79, *P* < 0.001), 130–160 mg/dL (3.4–4.1 mmol/L) (OR: 2.70, 95% CI: 1.92–3.880, *P* < 0.001), and ≥160 mg/dL (4.1 mmol/L) (OR: 4.38, 95% CI: 2.57–7.46, *P* < 0.001) but not in <100 mg/dL (2.6 mmol/L) (OR:3.02; 95% CI: 0.65–14.00, *P* = 0.16) (see Supplementary material online, *Figure S7*). The information of the treatment was reported in Supplementary material online, *Table S1*. In subgroup analysis according to different definitions of ‘premature’, increased level of Lp(a) was associated with significant higher risk of ASCVD among patients younger than 50 years (OR: 2.72; 95% CI: 2.16–3.41, *P* < 0.001) or younger than 60 years (OR:2.40, 95% CI:2.00–2.89, *P* < 0.001) (see Supplementary material online, *Figure S8*).

Subgroup analysis based on different cut-off levels indicated that the association between increased Lp(a) and premature ASCVD was significant when taking Lp(a) ≥ 30 mg/dL (72 nmol/L) (OR: 2.19, 95% CI: 1.75–2.74, *P* < 0.001), Lp(a) ≥ 50 mg/dL (110 nmol/L) (OR: 2.98, 95% CI: 1.94–4.58, *P* < 0.001), top quartiles (OR: 2.39, 95% CI: 1.55–3.68, *P* < 0.001), or top tertiles (OR: 2.22, 95% CI: 1.15–4.29, *P* = 0.02) as exposed but not for top quintiles (OR:1.47, 95% CI: 0.78–2.76, *P* = 0.23) (see Supplementary material online, *Figure S9*). The data of absolute value was reported in Supplementary material online, *Table S2*. To examine the potential influence of technique of Lp(a) measurements, further subgroup analysis was conducted, which revealed a pooled estimated OR of 1.86 (95% CI: 1.50–2.31, *P* < 0.001) for immunoturbidimetric method, 2.93 (95% CI: 2.15–3.98, *P* = 0.02) for IRMA, and 4.32 for ELISA (95% CI: 2.78–6.73, *P* < 0.001) (see Supplementary material online, *Figure S10*).

### Heterogeneity and sensitivity analyses

There was no significant heterogeneity in analyses of Lp(a) and overall ASCVD, whereas high heterogeneity was observed in analyses of Lp(a) and individual ASCVD. After excluding Li *et al.*,^29^ in which the important confounding factor of FH was ignored, the *I*² decreased to moderate from high, suggesting the unadjusted confounders could have contributed to the heterogeneity observed (see Supplementary material online, *Figure S11*). Subgroups stratified by study design, Lp(a) measurement approaches, baseline LDL-c level, definition of ‘premature’, gender, and race showed a substantial decline of heterogeneity, indicating the potential origins of heterogeneity (see Supplementary material online, *Figure S3∼S10*). Longenecker *et al.*^30^ are confounded due to dialysis and chronic kidney diseases (CKD). When excluded in the study, the result of the composite ASCVD remained significant. (see Supplementary material online, *Figure S12*) In addition, only Arnold *et al.*^6^ discussed the secondary prevention, while others discussed the primary prevention. After excluding the study, the result of the individual ASCVD remained significant (see Supplementary material online, *Figure S13*).

## Discussion

This comprehensive meta-analysis evaluated the associations between elevated Lp(a) and the risk of premature ASCVD; the main findings are the followings: (i) elevated Lp(a) is significantly associated with higher risk of composite premature ASCVD, especially for CAD and PAD, and amongst patients with FH and T2DM, and (ii) the association between Lp(a) and premature ASCVD was significant regardless of study design, gender, population characteristics (community or hospitalized), different premature definitions, and various Lp(a) measurement approaches.

Lp(a) is an LDL-c-like particle derived in the liver, which is covalently bound to apolipoprotein (apo) B100 by apo(a).^31^ It is the major carrier of oxidized phospholipid and promotes atherosclerotic plaque development through the pro-inflammatory, pro-thrombotic, and anti-fibrinolytic effects.^26,31–36^ In our meta-analysis, we found that genetically determined high Lp(a) is an independent predictor of premature ASCVD.

About 90% of the Lp(a) concentration is inherited and primarily determined by the LPA gene singly, so it shows more pronounced effect in younger patients, who are less affected by environmental risk factors accumulating with age.^37^ It is found that Lp(a) was a persuasive predictor of the composite ASCVD in young but not in old.^30^ For the individual ASCVD, the significant correlation was also observed in patients <60 years but not in those >60 years in the studies by Arnold *et al.*^6^ and Hanif *et al.*^38^ Rallidis *et al.*^39^ further found that a 10 mg/dL increase in Lp(a) was associated with 4% of higher risk of acute coronary syndrome in patients <45 years and 2% of higher risk in patients of 45–60 years. All studies above agreed that the correlation decreased with increasing age.

So far, there has been only one similar meta-analysis focusing on the impact of high Lp(a) on premature CAD. It is reported that Lp(a) concentration increased significantly in patients with premature CAD (standardized mean difference (SMD): 0.97, 95% CI: 0.52–1.42, *P* < 0.001, *I*² = 98%) compared with controls. But the results remained controversial because of relatively small case-control studies of moderate quality and high statistical heterogeneity.^40^ Compared with the previously published meta-analyses, our study has several strengths. Firstly, all observational studies meeting the criteria were included, so the impact of elevated Lp(a) on premature ASCVD in cohort studies and cross-sectional studies could be analysed. Secondly, most included studies were assessed as high quality; thus, the reliability of the results was guaranteed. Thirdly, we performed sensitivity analysis and subgroup analysis to reduce the heterogeneity. Fourthly, the influence of high Lp(a) on composite ASCVD, stroke, and PAD was reported, which was absent in the previous study.

It is worth noting that the correlation is not significant for ischaemic stroke. Ischaemic strokes are caused by several pathologies, including atherosclerotic vascular disease in large arteries, arteriolar disease in small arteries, and embolic disease caused by aorta or carotid artery atherosclerosis and heart disease, such as atrial fibrillation.^41^ Lp(a) levels of LAA stroke were significantly higher than those of the other stroke mechanisms. A two-sample Mendelian randomization analyses reported that reduction of Lp(a) levels was associated with lower risks for LAA stroke but not for any IS, cardioembolic stroke, or small vessel stroke. Arnold also found that elevated Lp(a) was independently associated with (LAA) ischaemic stroke aetiology but not with non-LAA ischaemic stroke.^6^ However, some of the included studies reporting premature ischaemic stroke neither identified the classification nor indicated evident arteriosclerotic disease in patients. It is possible that only LAA stroke is associated with elevated Lp(a) in young patients due to its machine of proatherosclerotic effect. We need more study with high quantity to determine the association for different subtypes of ischaemic stroke in young patients, and specific mechanisms should be clarified to evaluate the correlation between elevated Lp(a) and premature ischaemic stroke.

Familial hypercholesterolaemia is an inherited metabolic disease characterized by high levels of circulating LDL-c and is relatively prevalent in premature ASCVD patients.^42^ The current meta-analysis confirmed strong correlation between elevated Lp(a) and premature ASCVD in FH patients compared with the general population. Elevated Lp(a) is the key factor for risk stratification in the management of FH. Some guidelines suggested that Lp(a) should be incorporated into the genetic cascade testing of FH to identify family members in high risk.^43–45^ A research team at Fuwai Hospital found that in older patients comorbid with T2DM, the ASCVD risk associated with Lp(a) might further increase.^46^ And in this study, increased Lp(a) level is also a risk factor for premature ASCVD in T2DM patients. As for dialysis patients with CKD, in whom the Lp(a) was higher than others, the significant association between Lp(a) and premature ASCVD was revealed by Longenecker *et al.*^30^ Whether this finding applied to CKD patients at the early age remained to be confirmed, for the existing studies focusing on young people with CKD and dialysis were limited and the subgroup analysis failed. Arnold *et al.*^6^ reported significant relation between higher Lp(a) and recurrence stroke, but the subgroup analysis for secondary prevention population still needed more original studies.

Although Lp(a) concentration is predominantly determined by genetics, gender exerts an important influence.^47^ Therefore, subgroup analysis based on gender was conducted. There was an agreement that Lp(a) could predict ASCVD in men, but the prognostic value in women remained controversial.^4^ A Mendelian randomization study showed that correlation existed in both men and women,^48^ while Wild *et al.*^49^ reported only in men. We found that men could benefit from Lp(a) testing. As for women, although we reached a positive conclusion, significant heterogeneity might raise some question. The inconsistent findings might be due to various oestrogen levels of women included in different studies, and research for women stratified by menstruation and oestrogen replacement treatment is needed in the future to explore the influence on women.

Besides gender, ethnicity also have an impact on Lp(a) level, so we performed subgroup analysis for different races. Not only do they differ in Lp(a) concentration, patients among different races also show various population-attributable risk of elevated Lp(a) for MI in the populations of all ages, ranging from 0% in Africans to 9.5% in South Asians and modest in Caucasians (4.6%).^50^ Our meta-analysis showed significantly increased risk for premature ASCVD in Caucasians and South Asians with high Lp(a). South Asians and Caucasians could benefit from Lp(a) testing. In the review by Li *et al.*^29^ summarizing recent research regarding to Lp(a)-related studies in the Chinese population, it is recommended to test Lp(a) at least once in a lifetime. However, this study draws the opposite conclusion in young Chinese population, suggesting the necessity varied among Chinese in different age stratification. To present meaningful data on this topic, we look forward to large studies which analysed Lp(a) levels progressively for each decade of life to determine the parts of Chinese population in need of Lp(a) testing. Because the data about African-Caribbean patients were too little to conduct a meta-analysis, more studies are needed to provide sufficient information.

It’s found that high variability of Lp(a) levels could be considered an independent risk factor for increased post-percutaneous coronary intervention (PCI) C-reactive protein (CRP) level. The result of subgroup analysis of patients under 65 years remained consistent.^51^ In FH patients, Cao *et al.* found that high visit to visit variability of Lp(a) levels were associated with major adverse cardiovascular events (MACE). The repeated process of dissolution and crystallization of the cholesterol within coronary plaques caused by the variability of lipid might impair the plaque stability, thereby leading to plaque rupture and cardiovascular events.^52^ In contrast, a large, observational study found that difference between follow-up and baseline lipoprotein(a) molar concentration was not significantly associated with incident CAD.^53^ No consensus has been reached on this issue. In our meta-analysis, given the lack of related data in the original articles, we could not further investigate the association between Lp(a) variability with premature ASCVD. More study are needed to clarify the relationship between high variability of Lp(a) levels and cardiovascular disease.

Cumulative Lp(a) exposure, incorporating both the Lp(a) concentration and exposure duration into a single risk parameter, was an important predictive factor in the secondary prevention. Wang *et al.*^54^ found that higher level of cumulative Lp(a) exposure was related with poorer prognosis among individuals with pre-diabetes and T2DM. Due to the stability of Lp(a) level during the lifespan, in the individuals with higher Lp(a) level in the early age, high cumulative Lp(a) exposure tended to be reached faster, which explained the relationship between elevated Lp(a) and premature ASCVD from another perspective.

Premature ASCVD occurred more often in patients with elevated Lp(a) compared with those without when the baseline LDL-c ≥ 100 mg/dL (2.6 mmol/L). It is considered that Lp(a) contributed to a residual risk of ASCVD even at relatively low baseline LDL-c concentration of 100–130 mg/dL (2.6–3.4 mmol/L). However, when the threshold dropped further to 55 mg/dL (1.4 mmol/L), which was the LDL-c control target of patients with ASCVD recommended by the 2019 European Society of Cardiology (ESC)/European Atherosclerosis Society (EAS) guideline,^55^ the correlation no longer existed in older population.^56,57^ The mechanism of this unique association was not yet clear. It is found that the degradation of Lp(a) was partly mediated by the LDL receptors. High levels of LDL-c might occupy the receptors, competitively inhibiting the catabolism of Lp(a) and enhancing the biological effect of Lp(a). Patients with very low LDL-c levels tended to have high levels of activity of LDL receptors and a strong metabolic capacity for Lp(a). Although Lp(a) levels were high, they could be metabolized quickly and the biological effects were weakened.^57^ In adults at the early age, those with LDL-c below the cut-off of 2.6 mmol/L would not suffer from more often ASCVD for elevated Lp(a) according to the meta-analysis, providing a reference threshold for the prevention of early-onset ASCVD. The patients with combination of LDL-c ≥ 100 mg/dL (2.6 mmol/L) and Lp(a) elevations should be considered as the vulnerable ones and need to further reduce their LDL-c levels below 100 mg/dL (2.6 mmol/L). For only two study focused on patients with baseline LDL-c < 100 mg/dL (2.6 mmol/L), further studies were needed to clarify the LDL-c control target for young patients with high Lp(a).

Moreover, it is seemed that the association between Lp(a) levels and ASCVD events follows a linear pattern. Whether the results are significant might be related to the increased doses of Lp(a) in the exposed group compared with the control group. Lp(a) is often termed as a categorical variable through setting a threshold directly, which is more convenient in clinical practice. The risk of premature ASCVD is considered moderate at the level of 30–50 mg/dL (72–110 nmol/L), high above 50 mg/dL (110 nmol/L), and very high above 180 mg/dL (450 nmol/L).^58^ In this meta-analysis, significant association was observed when the ‘high Lp(a)’ was defined according to commonly used thresholds, 30 mg/dL (72 nmol/L) and 50 mg/dL (110 nmol/L). Among included studies, none reported outcomes in patients with Lp(a) level > 180 mg/dL, and we could not conduct a subgroup analysis focusing on these patients in very high risk, which is one of the limitations of our study. Moreover, exploring the prognostic effects of very high levels of Lp(a) in patients with prior ASCVD may be of little significance. Berman *et al.* conducted a retrospective cohort study and found that the there was a plateau around 70% in risk for patients with prior ASCVD when analysing the association between Lp(a) and ASCVD, whereas there was a linear association between Lp(a) and risk among patients without ASCVD. For this situation, they explained that in the patients with baseline ASCVD, which already had higher absolute risk of CVD events and were treated with optimal preventive therapies usually, extremely elevated Lp(a) (above the 90th percentile) might have less effect on the future ASCVD risk. The threshold for risk assessment was 53 mg/dL (112 nmol/L) in secondary prevention, while the threshold was 102 mg/dL (216 nmol/L) in primary prevention, suggesting that elevated Lp(a) should be defined differently for different populations for optimal risk prediction and clinical management.^59^

However, it is strange that people with top quintile Lp(a) did not show higher risk of premature ASCVD, but people with top quartile and tertile did. The study from Shi *et al.*^9^ should be rebuked for it focused on Chinese people, in whom the association was not significant. Moreover, due to the small total number of included studies, the result of the subgroup analysis was less reliable.

The findings of the meta-analysis could be clinically relevant. The patients with higher Lp(a) might suffer from ASCVD earlier, so the Lp(a) testing for reclassification in people who are borderline between moderate and high risk^60^ and the preventive measures to lower Lp(a) should be taken in time, especially in males, females, Caucasians, South Asians, FH patients, and T2DM patients. It is recommended in the guideline that measurement of Lp(a) is a routine at least once in a lifetime.^55,61^ Young patients with higher Lp(a) levels should be given earlier appointment for decisive tests like computed tomography angiography or invasive angiography, regardless of their history of T2DM or FH.^38^ For the approach to reducing Lp(a) level, existing data have indicated that intensification of treatment, such as the proprotein convertase subtilisin/kexin type 9 (PCSK9) inhibitors, evolocumab, and alirocumab, could reduce Lp(a) levels by about 20–30%, providing great risk reduction for cardiovascular risk.^26,58^ In addition, lipoprotein apheresis could lower Lp(a) immediately.^62^ Novel drugs under development such as RNA-targeting therapy was still in the early development stage.^63^ Moreover, a more aggressive management of lifestyle modifications and reduction of elevated LDL-c levels, especially with statins, due to the synergistic effects of Lp(a) and LDL-c, was needed.^27,39^

## Limitation

Several potential limitations should be noted in our meta-analysis. Firstly, only articles published on PubMed and Embase were included, which might lead to missing articles that were not indexed in these search engines. Secondly, in consideration of the characteristics of the included studies, Lp(a) concentration was analysed as a continuous {per one unit or per 1 SD increase of Lp(a) or log [Lp(a)]} and categorical variable (tertile, quartile, quintile, or specific thresholds), resulting in failure of pooling all relative studies together. Thirdly, most studies included in our studies were retrospective studies and cross-sectional studies, which might have more recall bias. Fourthly, publication bias in meta-analyses was examined by checking for asymmetry in a funnel plot, which was determined subjectively. Fifthly, in subgroup analyses based on races, we failed to provide data about other races such as data for the lack of information. Finally, certain analysis contained only two or three studies, which were relatively few, and more studies were needed to increase the reliability of the results.

## Conclusions

Our meta-analysis supports that elevated Lp(a) concentration, which is genetically determined, can predict both composite and individual ASCVD in young patients. The presence of high Lp(a) concentration indicates prospective evaluation and validation as a clinical risk factor in premature ASCVD in Caucasians, South Asians, FH population, and patients with the baseline LDL-c level ≥ 100 mg/dL (2.6 mmol/L).

## Supplementary Material

oeae031_Supplementary_Data

## Data Availability

The data in this study could be made available upon reasonable request to the corresponding author.

## Lead author biography

Tong Liu, FESC, FHRS, ISNE-F, is a professor and doctoral supervisor at Tianjin Medical University. He is the Director of the Department of Cardiology, Second Hospital of Tianjin Medical University, and Director of Tianjin Institute of Cardiology. He is mainly engaged in basic and clinical research cardio-oncology and atrial fibrillation. He is a Committee Member of the 1st and 2nd Integrated Cardiology Branch of the Chinese Anti-Cancer Association and a Youth Committee member of ISE. He is Editor in Chief of *Current Cardiology Reviews* (SCI) and an Editorial Board Member of *Cardio-Oncology* (SCI), *PACE*, *BMC Cardiovascular Disorders*, and *Journal of Clinical Medicine*.
