# Supplementary material for: Association between lipoprotein(a) and premature atherosclerotic cardiovascular disease: a systematic review and meta-analysis
Source: Eur Heart J Open. 2024 Apr 26;4(3):oeae031. doi: 10.1093/ehjopen/oeae031 (PMC11086656; doi:10.1093/ehjopen/oeae031)
Supplement: oeae031_Supplementary_Data [file oeae031_supplementary_data.doc]

# **Supplementary Material**

**Supplementary Material 1:** Search strategy

**Supplementary Material 2:** Funnel plots (Figure S1)

**Supplementary Material 3:** Results for continuous variables (Figure S2)

**Supplementary Material 4:** Subgroup analyses (Figure S3~S10)

**Supplementary Material 5:** Sensitivity analyses (Figure S11~S13)

**Supplementary Material 6:** Added information (Table 1~2)

# **Supplementary Material 1: Search Strategy - 26/10/2023**

PubMed:

((((((((((cardiovascular[Title/Abstract]) OR (cardiac[Title/Abstract])) OR (heart[Title/Abstract])) OR (coronary[Title/Abstract])) OR (myocardial[Title/Abstract])) OR (peripheral arterial diseases[Title/Abstract])) OR (peripheral artery diseases[Title/Abstract])) OR (ischemic stroke[Title/Abstract])) OR (atherosclerosis[Title/Abstract])) OR (atherogenesis[Title/Abstract])) AND (((("Lipoprotein Lp(a)"[Title/Abstract]) OR ("Lipoprotein(a)"[Title/Abstract])) OR ("Lipoprotein a"[Title/Abstract])) OR ("Lipoprotein(a-)"[Title/Abstract]))

Embase:

#1 ’cardiovascular’:ab,ti OR ‘cardiac’:ab,ti OR ‘heart’:ab,ti OR ‘coronary’:ab,ti OR ‘myocardial’:ab,ti OR ‘peripheral arterial diseases’:ab,ti OR ‘peripheral artery diseases’:ab,ti OR ‘ischemic stroke’:ab,ti OR ‘atherosclerosis’:ab,ti OR ‘atherogenesis’:ab,ti

#2 'lipoprotein a'/exp OR 'lipoprotein lp(a)':ab,ti OR 'lipoprotein(a)':ab,ti OR 'lipoprotein a':ab,ti OR 'lipoprotein(a-)':ab,ti

#3 ‘premature’:ab,ti OR ‘young’:ab,ti OR ‘younger’:ab,ti OR ‘youth’:ab,ti OR ‘untimely’:ab,ti OR ‘middle aged’:ab,ti OR ‘early’:ab,ti OR ‘earlier’:ab,ti OR ‘earliest’:ab,ti OR ‘less than’:ab,ti OR ‘before’:ab,ti OR ‘under’:ab,ti OR ‘age’:ab,ti

#1 AND #2 AND #3

**Supplementary Material 2: Funnel plots**


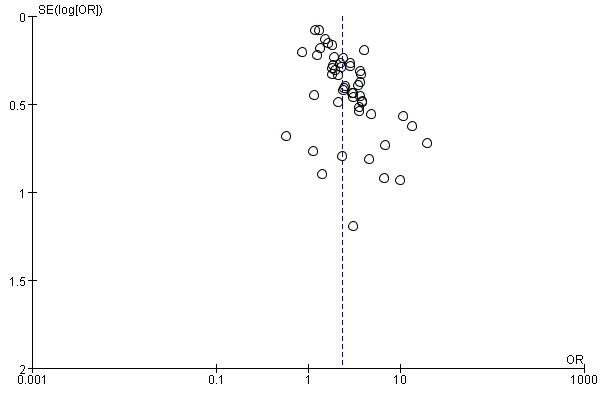


**Figure S1.** **Funnel plots of the meta-analysis.** Funnel plot for elevated Lp(a) as a categorical variable and risk of premature ASCVD. Abbreviations: ASCVD: Arteriosclerotic cardiovascular disease; CI: Confidence interval; Lp(a): Lipoprotein(a); OR: odds ratio; SE: standard error

**Supplementary Material 3: Results for continuous variables**


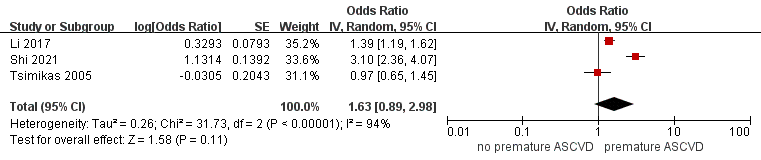
**A**


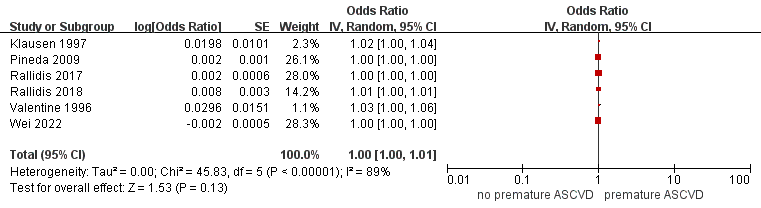
**B**

**Figure S2** Association between elevated Lp(a) and premature ASCVD for per 1 unit increase of log[Lp(a)] (A) and for per 1 unit increase of Lp(a) (B).

Abbreviations: ASCVD: Arteriosclerotic cardiovascular disease; Lp(a): Lipoprotein(a); CI: Confidence interval; OR: odds ratio; SE: standard error

**Supplementary Material 4: Subgroup analyses**


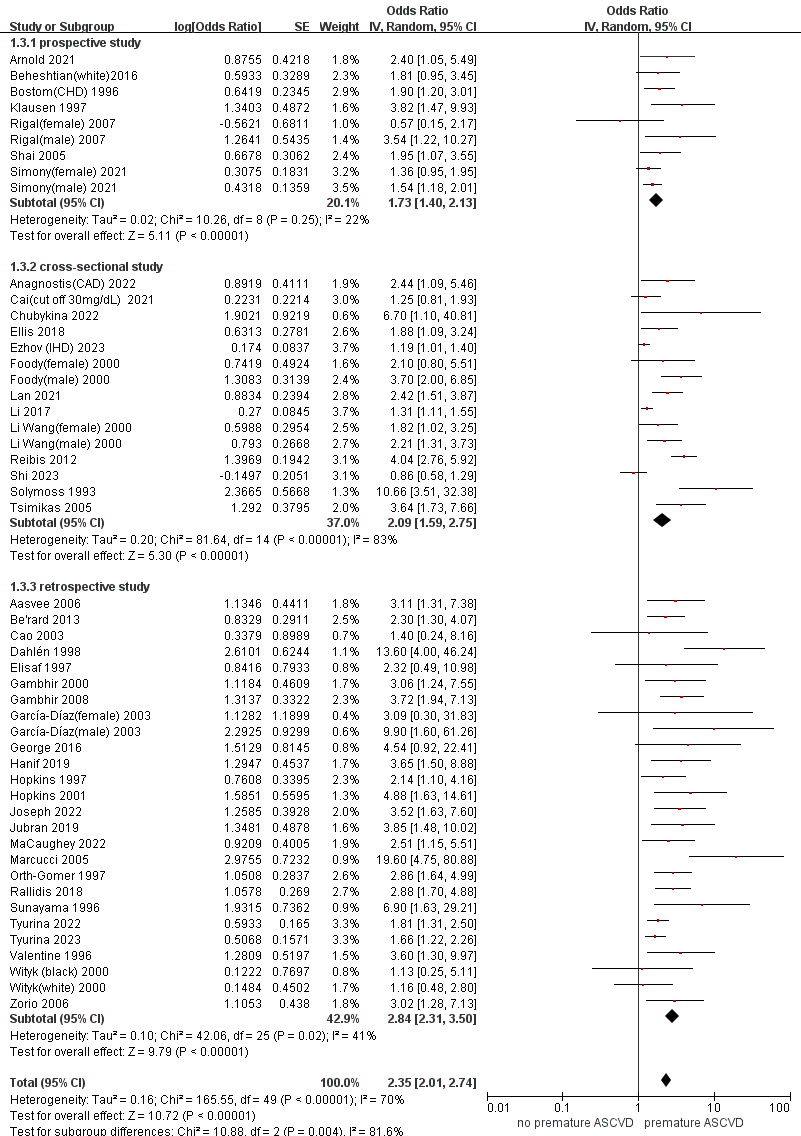


**Figure S3.** Subgroup analysis according to study design when Lp(a) was analyzed as a categorical variable.

Abbreviations: ASCVD: Arteriosclerotic cardiovascular disease; Lp(a): Lipoprotein(a); CI: Confidence interval; OR: odds ratio; SE: standard error


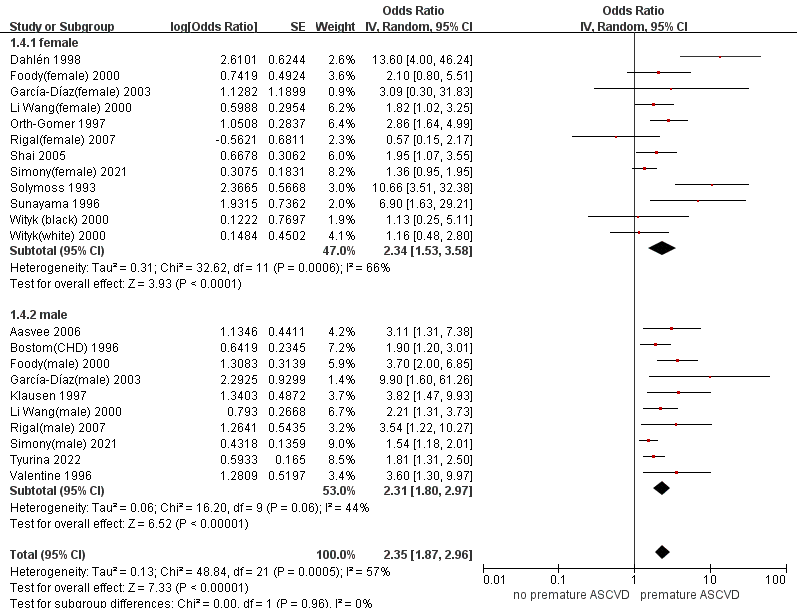


**Figure S4.** Subgroup analysis according to gender when Lp(a) was analyzed as a categorical variable.

Abbreviations: ASCVD: Arteriosclerotic cardiovascular disease; Lp(a): Lipoprotein(a); CI: Confidence interval; OR: odds ratio; SE: standard error


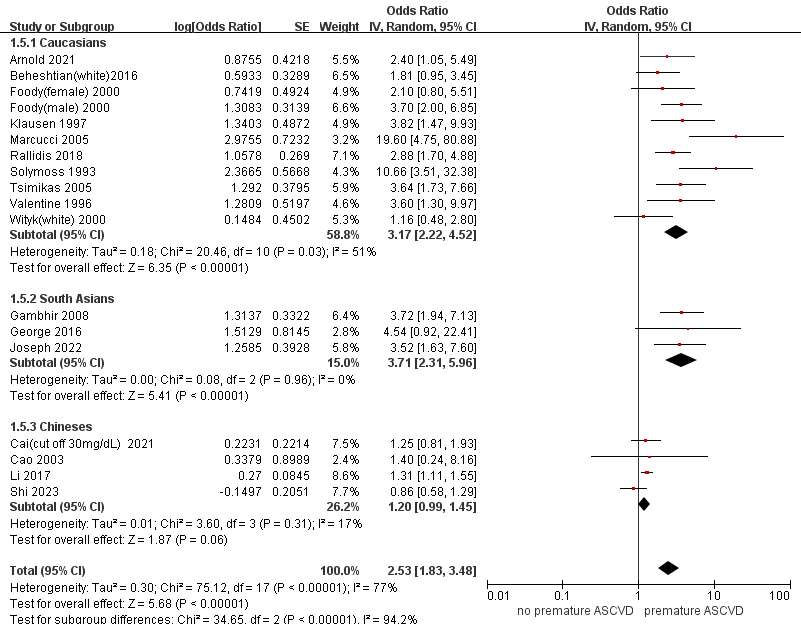


**Figure S5.** Subgroup analysis according to race when Lp(a) was analyzed as a categorical variable.

Abbreviations: ASCVD: Arteriosclerotic cardiovascular disease; Lp(a): Lipoprotein(a); CI: Confidence interval; OR: odds ratio; SE: standard error


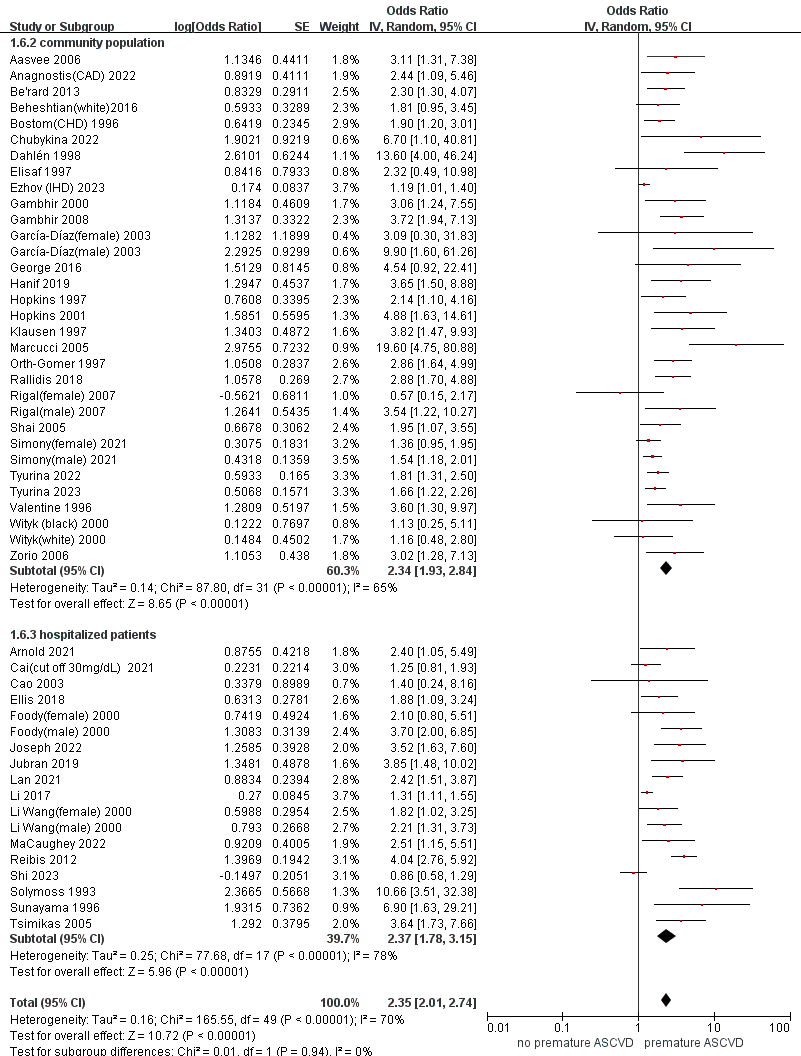


**Figure S6.** Subgroup analysis according to population characteristics when Lp(a) was analyzed as a categorical variable.

Abbreviations: ASCVD: Arteriosclerotic cardiovascular disease; Lp(a): Lipoprotein(a); CI: Confidence interval; OR: odds ratio; SE: standard error


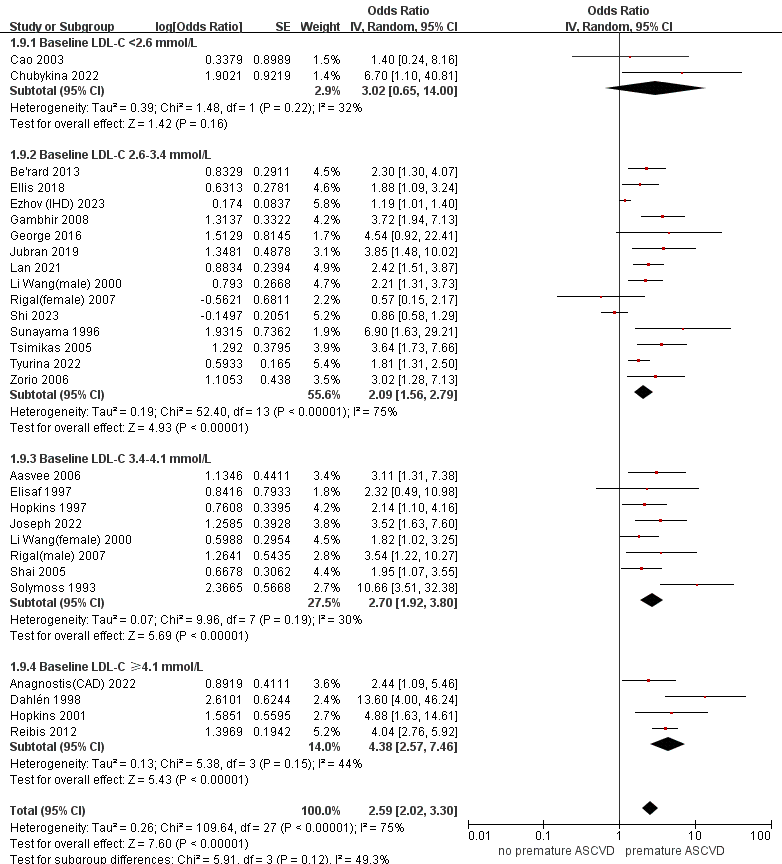


**Figure S7.** Subgroup analysis according to baseline LDL-c level when Lp(a) was analyzed as a categorical variable.

Abbreviations: ASCVD: Arteriosclerotic cardiovascular disease; LDL-c: Low density lipoprotein cholesterol; Lp(a): Lipoprotein(a); CI: Confidence interval; OR: odds ratio; SE: standard error


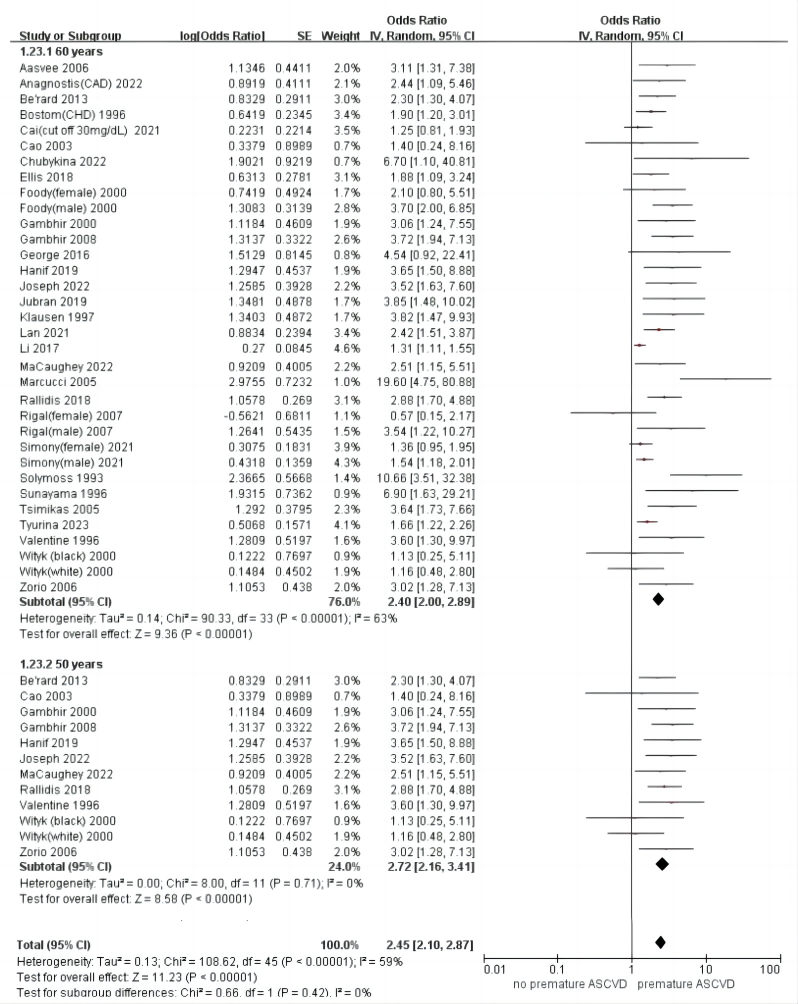


**Figure S8.** Subgroup analysis according to definition of “premature” when Lp(a) was analyzed as a categorical variable.

Abbreviations: ASCVD: Arteriosclerotic cardiovascular disease; Lp(a): Lipoprotein(a); CI: Confidence interval; OR: odds ratio; SE: standard error


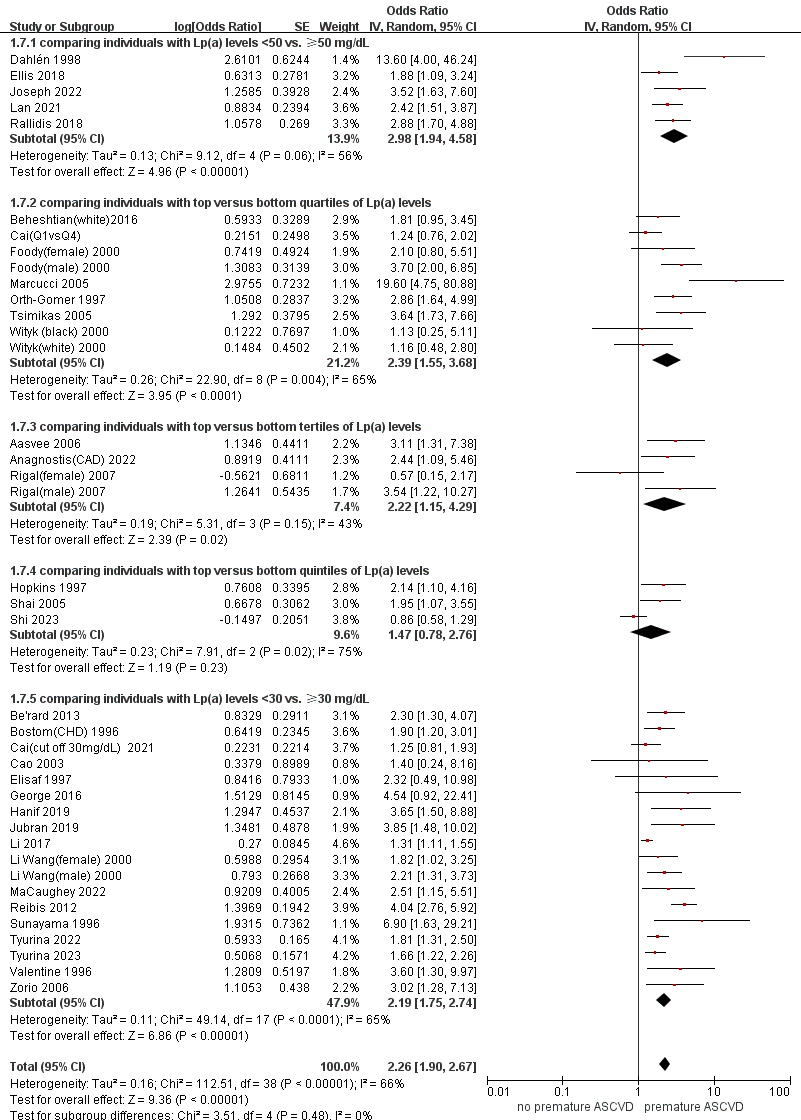


**Figure S9.** Subgroup analysis according to different cut-off levels when Lp(a) was analyzed as a categorical variable.

Abbreviations: ASCVD: Arteriosclerotic cardiovascular disease; Lp(a): Lipoprotein(a); CI: Confidence interval; OR: odds ratio; SE: standard error


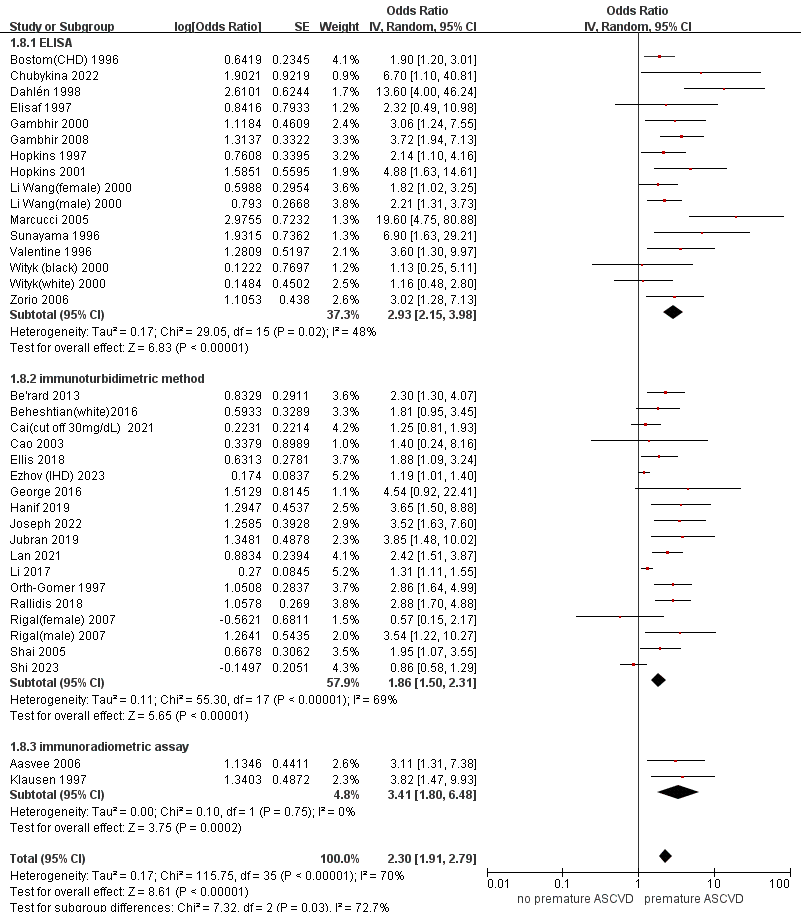


**Figure S10.** Subgroup analysis according to technique of measurement when Lp(a) was analyzed as a categorical variable.

Abbreviations: ASCVD: Arteriosclerotic cardiovascular disease; Lp(a): Lipoprotein(a); CI: Confidence interval; ELISA: Enzyme linked immunosorbent assay OR: odds ratio; SE: standard error

**Supplementary Material 5: Sensitivity analyses**


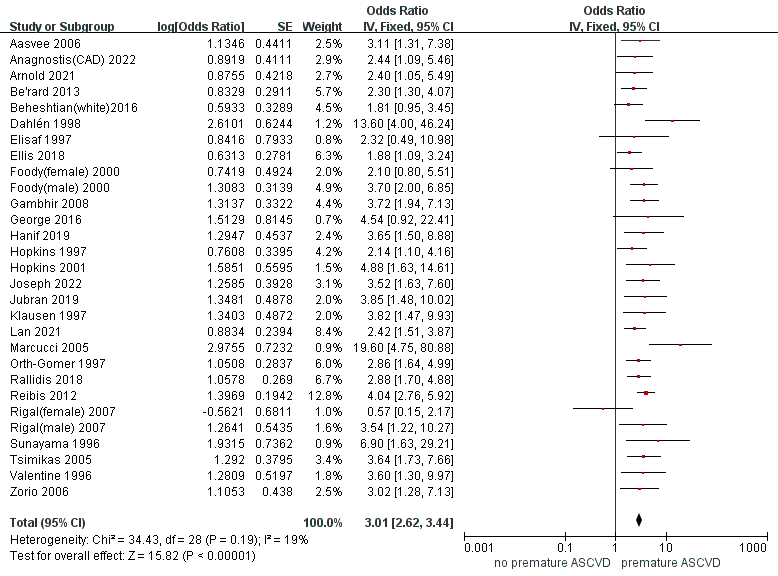


**Figure S11. The results of sensitivity analysis** elevated Lp(a) as a categorical variable and risk of premature ASCVD.

Abbreviations: ASCVD: Arteriosclerotic cardiovascular disease; Lp(a): Lipoprotein(a); CI: Confidence interval; OR: odds ratio; SE: standard error


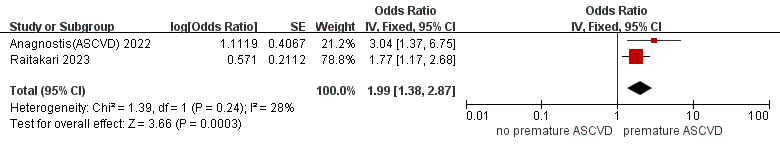


**Figure S12. The results of sensitivity analysis** elevated Lp(a) as a categorical variable and risk of the composite premature ASCVD.

Abbreviations: ASCVD: Arteriosclerotic cardiovascular disease; Lp(a): Lipoprotein(a); CI: Confidence interval; OR: odds ratio; SE: standard error


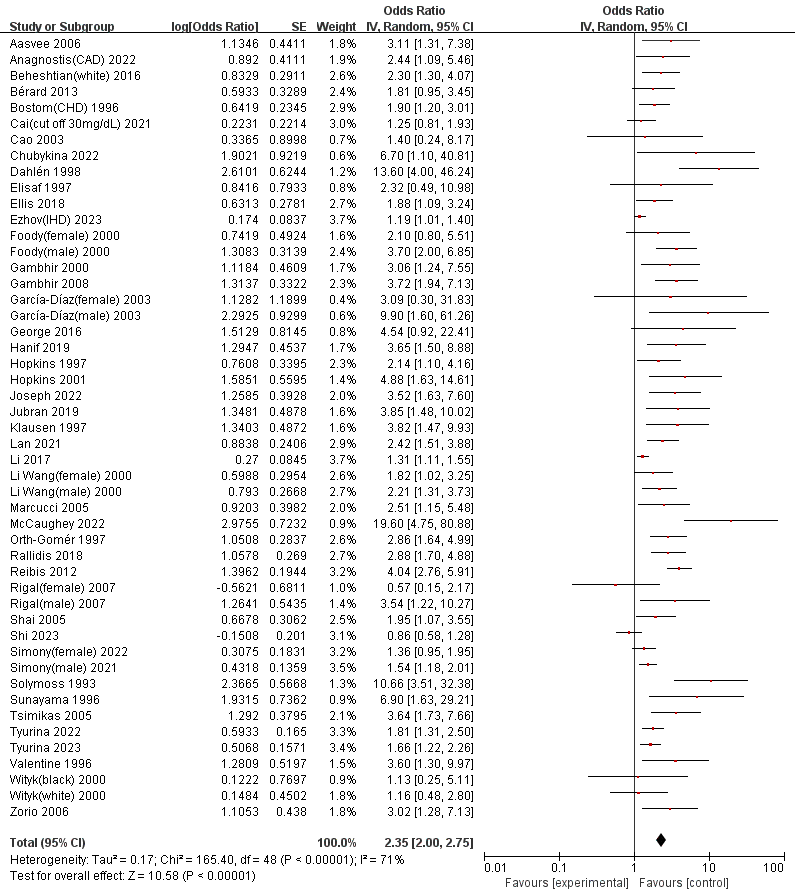


**Figure S13. The results of sensitivity analysis** elevated Lp(a) as a categorical variable and risk of premature ASCVD.

Abbreviations: ASCVD: Arteriosclerotic cardiovascular disease; Lp(a): Lipoprotein(a); CI: Confidence interval; OR: odds ratio; SE: standard error

**Supplementary Material 6: Added information**

Table 1.Use of drugs potentially influencing on the blood lipid levels.

| Study and published year | Baseline LDL-c levels (mmol/L) | Treatment | Number of patients treated（%） |
| --- | --- | --- | --- |
| Chubykina 2022 | <2.6 | Statins | 73(44.5) |
| Bérard 2013 | 2.6-3.4 | Statins | 31(10.2) |
|  |  | Statins+antiplatelet | 25(8.3) |
| Ellis 2018 | 2.6-3.4 | Statins | 121(38.3) |
| Jubran 2019 | 2.6-3.4 | Statins | 65(49) |
| Lan 2021 | 2.6-3.4 | Statins | 88(59.9) |
| Rigal(female)2007 | 2.6-3.4 | Statins | 7(16.6) |
| Sunayama 1996 | 2.6-3.4 | Lipid-lowering drugs | 61(17.2) |
| Tsimikas 2005 | 2.6-3.4 | Statins | 142 (28) |
| Tyurina 2022 | 2.6-3.4 | Statins | 169(74.1) |
| Li Wang (male) 2000 | 2.6-3.4 | Lipid-lowering drugs | 303(30.5) |
|  |  | β-blockers | 379(38.1) |
| Elisaf 1997 | 3.4-4.1 | β-blockers | 59(27.8) |
|  |  | Diuretics | 33(15.6) |
|  |  | Calcium channel  blockers | 35(16.5) |
|  |  | Antidiabetic | 7(3.3) |
| Hopkins 1997 | 3.4-4.1 | Lipid-lowering drugs | 98(29.3) |
| Rigal (male) 2007 | 3.4-4.1 | Statins | 23(19.8) |
| Li Wang (female) 2000 | 3.4-4.1 | Lipid-lowering drugs | 92(29.4) |
|  |  | β-blockers | 113(36.1) |
|  |  | Estrogen replacement | 249(27) |
|  |  | Insulin | 178(19.3) |
|  |  | Oral diabetic medication | 183(19.9) |
| Solymoss 1993 | 3.4-4.1 | Thiazides | 21(12.1) |
|  |  | β-blockers | 52(29.9) |
|  |  | Estrogen replacement | 13(7.5) |
|  |  | Thyroxine replacement | 11(6.3) |
| Hopkins 2001 | 3.4-4.1 | Lipid-lowering drugs | 115(43.9) |

Abbreviations: LDL-c: Low density lipoprotein cholesterol

Table 2. Data about corresponding absolute values when evaluating tertile/quartile/quintile

| Study and published year | Cut-off levels | Absolute values |
| --- | --- | --- |
| Aasvee 2006 | tertile | 1^st^ <95mg/dL |
|  |  | 2^nd^ 95-326mg/dL |
|  |  | 3^rd^>326mg/dL |
| Anagnostis 2022 | tertile | 1^st^ 6.4mg/dL(3.0-9.7)* |
|  |  | 2^nd^ 22.4mg/dL(16.0-29.1)* |
|  |  | 3^rd^ 77.0mg/dL(55.0-102.0)* |
| Rigal 2007 | tertile | 1^st^ <0.07g/L |
|  |  | 2^nd^ 0.07-0.29g/L |
|  |  | 3^rd^ ≥0.30g/L |
| Beheshtian 2016 | quartile | NA |
|  |  | NA |
|  |  | NA |
|  |  | NA |
| Cai 2019 | quartile | 1^st^ <51mg/L |
|  |  | 2^nd^ 51-108mg/L |
|  |  | 3^rd^ 108-215mg/L |
|  |  | 4^th^ ≥215mg/L |
| Foody 2000 | quartile | 1^st^ ≤5mg/dL |
|  |  | 2^nd^ 6-19mg/dL |
|  |  | 3^rd^ 20-44mg/dL |
|  |  | 4^th^ ≥45mg/dL |
| Marcucci 2005 | quartile | NA |
|  |  | NA |
|  |  | NA |
|  |  | NA |
| Orth-Gomer 1997 | quartile | 1^st^ 0.01-0.06g/L |
|  |  | 2^nd^ 0.07-0.14g/L |
|  |  | 3^rd^ 0.15-0.30g/L |
|  |  | 4^th^ >0.30g/L |
| Tsimikas 2005 | quartile | NA |
|  |  | NA |
|  |  | NA |
|  |  | NA |
| Wityk 2000 | quartile | NA |
|  |  | NA |
|  |  | NA |
|  |  | NA |
| Hopkins(female)1997 | quintile | 1^st^ <1.8mg/dL |
|  |  | 2^nd^ 1.8-4.3mg/dL |
|  |  | 3^rd^ 4.4-8.0mg/dL |
|  |  | 4^th^ 8.1-26.0mg/dL |
|  |  | 5^th^ >26.0mg/dL |
| Hopkins(male) 1997 | quintile | 1^st^ <3.1mg/dL |
|  |  | 2^nd^ 3.1-6.7mg/dL |
|  |  | 3^rd^ 6.8-14.7mg/dL |
|  |  | 4^th^ 14.8-32.5mg/dL |
|  |  | 5^th^ >32.5mg/dL |
| Shai 2005 | quintile | 1^st^ 0.0-0.09μmol/L |
|  |  | 2^nd^ 0.09-0.20μmol/L |
|  |  | 3^rd^ 0.20-0.36μmol/L |
|  |  | 4^th^ 0.36-1.01μmol/L |
|  |  | 5^th^ 1.01-6.30μmol/L |
| Shi 2023 | quintile | 1^st^ <60.0mg/L |
|  |  | 2^nd^ 60.0-120.0mg/L |
|  |  | 3^rd^ 120.0-201.0mg/L |
|  |  | 4^th^ 201.0-385.8mg/L |
|  |  | 5^th^ >385.8mg/L |

*Data are presented as median (interquartile range)
